# Late onset psychosis treatment with adjunctive medicines

**DOI:** 10.3389/fpsyt.2023.1319891

**Published:** 2023-12-21

**Authors:** Irina Boksha, Olga Savushkina, Vladimir Sheshenin, Elena Tereshkina, Tatyana Prokhorova, Valeriya Pochueva, Gulnur Burbaeva

**Affiliations:** Mental Health Research Centre, Moscow, Russia

**Keywords:** late onset psychosis, antipsychotic, adjunctive medicines, blood biochemical parameters, correlative analysis

## Abstract

**Background:**

A number of studies have shown the feasibility of using adjunctive drugs in late onset psychosis (LOP).

**Aim:**

Testing hypothesis that among LOP people treated with antipsychotics and antidepressants, basing on certain clinical characteristics a subgroup of patients might be distinguished, for whom adjunctive therapy is advantageous. This subgroup might be identified by measurement of blood biochemical parameters.

**Methods:**

59 in-patients with LOP, treated neuroleptics and antidepressants, were included, and followed in real clinical practice. Database containing demographic, clinical data (scores by PANSS, CDSS, CGI-S, HAMD-17), prescribed therapy, adverse effects of antipsychotic and antidepressant treatment, and blood biochemical parameters (enzymatic activities of glutamate- and glutathione metabolism enzymes in platelets and erythrocytes) at baseline and after the treatment course was created.

**Results:**

Three groups of patients (Gr1, Gr2, and Gr3), based on the adjunctive therapy usage were identified: Gr1 (n = 16) was without adjunctive therapy, two other groups (Gr2 and Gr3) were with adjunctive medicines, such as 2-ethyl-6-methyl-3-hydroxypyridine succinate (EMHS; Gr2, *n* = 20), or other drugs, such as citicoline, cerebrolysin, cortexin, actovegin, gliatilin (choline alfoscerate; Gr3, *n* = 23). The enzymatic activities were assessed also in the matched control group (*n* = 38). In all three patient groups, as compared with controls, activity of erythrocyte glutathione reductase was decreased at baseline and after the treatment course. In Gr2, unlike Gr1 or Gr3, there was a significant decrease in baseline glutamate dehydrogenase and glutathione-S-transferase activities. Certain clinical criteria were also elucidated for prescription of EMHS as adjunctive therapy for patients of Gr2. Glutamate dehydrogenase and glutathione-S-transferase activities returned closer to control levels after the treatment course in Gr2, unlike Gr1, where they declined yet more after psychotropic treatment without adjunctive medicine. Different significant links between biochemical parameters and scores by clinical scales were observed in Gr1, Gr2, and Gr3, some having predictive value for evaluation of antipsychotic treatment efficacy.

**Conclusion:**

We demonstrate the validity of adjunctive neuroprotective medicines’ usage in addition to antipsychotic and antidepressant therapy in distinct subgroups of patients suffering with LOP, especially those who have prominent side effects accompanying their psychotropic treatment. Returning of biochemical parameters to control range following the treatment course observed in patients of the subgroup treated with adjunctive EMHS is evidence for their metabolism normalization.

## 1 Introduction

Psychotic disorders usually occur in adolescence and early adulthood. However, a significant portion of patients have a first psychotic episode in their 40s or older (referred to as late onset psychoses, LOP) (1). Due to the increase in older people in the general population, and insufficient knowledge of etiology, pathogenesis and treatment of mental disorders in older people, the LOP research is increasingly becoming important.

LOP remains one of the most controversial psychiatric syndromes: there are conflicting diagnostic approaches to LOP, and their differentiation from onset of neurodegenerative diseases makes it difficult to study them. The authors of an updated systematic review (2) covering 27 original clinical studies of LOP as well as psychoses with very late onset (VLOSLP; 2000–2019) noted that empirical studies of LOP are not well described in the current literature, and inconsistencies in published results may be due to a lack of uniformity in the definitions of LOP and VLOSLP. There is no consensus still on the definition of non-organic, non-affective psychoses at a later age. It is, therefore, difficult to analyze the available data due to the heterogeneity of clinical groups not only in terms of research methods, but also in terms of inclusion criteria.

In Russia (Mental Health Research Centre), we adhere to the definition given in 2000 based on the consensus statement of an international group of experts, late-onset schizophrenia-like psychosis (illness onset after 40 years of age, further we use the LOP abbreviation) and very-late-onset schizophrenia-like psychosis (VLOSLP, onset after 60 years) (2–4). Comprehensive assessment of the risks and benefits of pharmacological treatment is named as the focus of prospective research on this topic (2, 5), and it is on this item that we are focusing our attention in this paper.

The questions of the role of biological (biochemical) factors both in the development of LOP and in predicting the response to therapy of LOP remain unexplored. Cerebrovascular and somatic pathology typical for older patients necessitates polypharmacy which may result in unwanted side-effects and complications (5). Adjunctive drugs possessing neuroprotective and antioxidant effect are advantageous, and their use in mental disorders (i.e., schizophrenia, affective disorders) increases the efficacy of psychotropic pharmacotherapy, in particular by reducing the resistance and diminishing its side-effects, especially in old age (6).

One of neuroprotective antioxidant compounds, 2-ethyl-6-methyl-3-hydroxypyridine succinate (EMHS), or Mexidol, induces cerebral mitochondriogenesis in aging (7), and inhibits neuronal excitotoxicity *in vitro* (8). EMHS has been successfully used in complex therapy in neurology (i.e., ischemic brain lesions), infectious pathology (including rehabilitation after COVID-19) (9, 10) and psychiatry, including schizophrenia (11, 12) and old age psychiatry (13). EMHS is well tolerated, and its ability to prevent or alleviate side effects of antipsychotics, such as tardive dyskinesia, has been reported (11). However, EMHS use in schizophrenia, especially in LOP, is challenging, due to stimulating effect, it can be favorable only for some patients. Schizophrenic patients, especially those with LOP, are known to vary in their clinical and biochemical parameters and may be stratified according with their blood biochemistry parameters, related to antioxidant and glutamate metabolism, enabling identifying those patients who will benefit from EMHS (14). Such a stratification could enable more precise and effective usage of antipsychotic preparations and adjunctive therapy.

Other neuroprotective compounds such as citicoline, cerebrolysin, cortexin, actovegin, gliatilin (choline alfoscerate) have also been successfully used in neurology and psychiatry and are referred to as “metabolic” (15). Cortexin, cerebrolysin and actovegin contain a complex of natural biologically active substances that protect neurons, promote neuroplasticity and neurogenesis, improve energy metabolism and brain trophism in experimental animal models (16). A review summarizing experience of citicoline usage in neurological disorders highlights advantages of this medicine against cognitive impairment (17). Cerebrolysin has been used earlier in schizophrenia patients with an abundance of negative symptoms (18), whereas cortexin was used in cognitive impairment in behavioral disorders (19). There are several trials demonstrating advantages of actovegin in cognitive impairments accompanying ischemic stroke (20–22). Gliatilin (choline alfoscerate) has also been successfully introduced in neurological and psychiatric practice (23, 24).

The aim of current observational cross-sectional study was to test the hypothesis that among the heterogeneous population of LOP patients, there is(are) a subgroup(s) for which the treatment with medicines adjunctive to antipsychotic therapy is effective in relation to overcoming the side effects related to their psychotropic medication. These subgroups of patients might have distinct biochemical features discriminating them from other LOP patients.

## 2 Materials and methods

### 2.1 Clinical design, patients and clinical assessments

We conducted an open non-randomized clinical and biological observational cross-sectional study in real clinical practice. The study was approved by the local Ethical Committee of the Mental Health Research Centre (the Document No 8, at 26.02.2019). All participants provided an Informed consent. The study was carried out in accordance with The Helsinki Agreement of the World Medical Association (as amended in 1975/2000).

The clinical group consisted of 59 patients (56 females, 3 males), age 45–89 years (median age 66 [58; 75]), with a median age of LOP onset 55 [45; 69] years. They were all diagnosed as schizophrenia spectrum disorders, such as “schizophrenia,” “schizoaffective disorder,” “other persistent delusional disorders,” or “organic delusional (schizophrenia-like) disorder” by ICD-10 (F20, F25, F22.8, F06.2, respectively) (4). Exclusion criteria were as follows: drug-abuse, severe acute somatic disorders, dementia or overall score on the MMSE ≤ 25 at the end of the antipsychotic and antidepressant treatment course, organic brain impairment (tumor, infection, and trauma). Some patients had CT/MRI brain scans.

All enrolled patients were in-patients hospitalized in the Mental Health Research Center during 2019–2022. Clinical assessments were done twice: at baseline, when hospitalized, and after 4 weeks of treatment course. Mental state was assessed using clinical psychometric standard scales. The severity of psychosis was assessed using positive and negative syndrome scale (PANSS) (25), and clinical global impression scale-severity (CGI-S) (26). Calgary Depression Scale for Schizophrenia (CDSS) (27), and Hamilton Depression Rating Scale (HAMD-17, or HDRS) (28) were used for depression assessment. Cognitive function was assessed using Mini-Mental State Examination (MMSE) scale (29). The standard treatment course lasted 28 days.

#### 2.1.1 Treatment

All 59 patients were treated with antipsychotics, 48 of 59 patients received antidepressants additionally to antipsychotics (when depression symptoms were present), and 33 patients were treated with tranquilizers, as needed. All these medicines were chosen taking into account psychopathological and neurological indications. Individual selection of doses corresponded to clinical recommendations. Atypical and typical antipsychotics were used for the treatment, the drugs were applied in moderate therapeutic doses, usually well tolerated by the patients, with olanzapine (*n* = 30) and haloperidol (*n* = 18) being the most frequently prescribed antipsychotics. As for antidepressants, fluvoxamine (*n* = 25), sertraline (*n* = 16), and duloxetine (*n* = 10) were the most frequently prescribed due to the severity of depressive symptoms in the structure of psychosis, and clonazepam (*n* = 10) was the most frequently prescribed medicine for tranquilization.

As for adjunctive therapy, patients receiving EMHS (*n* = 20) were separated into a distinct subgroup, they were regarded apart from the patients receiving other adjunctive medicines. This was done since among all the applied adjunctive medicines EMHS alone possesses prominent antioxidant properties, whereas other medicines were assigned as nootropics (30). At the beginning of the antipsychotic therapy, EMHS was prescribed based on a clinician’ own previous psychiatric experience in older LOP patients (31). Thus, EMHS was prescribed when prominent side-effects caused by antipsychotics in the form of extrapyramidal symptoms were present and recorded by clinicians during previous experiences of treatment courses, or in the cases with elevated risk factors to the side-effects, such as cerebrovascular diseases, comorbid somatic pathology, that can affect the psychotropic treatment. Whereas the psychosis in the patient was less acute than in Gr1 or Gr3 patients (31). Neuroprotectants were prescribed for patients who complained weakness, cognitive impairment (cerebrolysin, cortexin, citicoline, choline alfoscerate), or who were with the higher risk of metabolic syndrome, diabetes, or orthostatic hypotension (actovegin).

Adjunctive medicines were applied as intravenous infusions, except cortexin, and the medicines’ doses were as follows: EMHS (*n* = 20)—200–300 mg per day (total 10–14 infusions), actovegin (*n* = 6)—400 mg; for citicoline (*n* = 7)—1,000 mg; for cerebrolysin (*n* = 5)—2,152 mg per day for 10 days, for choline alfoscerate—1,000 mg per day for 10 days. Cortexin (*n* = 5) was injected intramuscularly, 10 mg per day, for 10 days.

Psychometric assessments and blood biochemical parameters were estimated at baseline and at 28-th day of the treatment course. CDSS scores were estimated only at baseline (Table 1).

**Table 1:** Demographic and clinical data for Gr1, Gr2, and Gr3 groups of patients.

Parameter	Patient groups			Mann–Whitney test, *p*-level
	Gr1 (*n* = 16)	Gr2 (*n* = 20)	Gr3 (*n* = 23)	
Age, years	63 [56; 71]	65 [59; 70]	69 [61; 80]	*p* > 0.05
Disease duration, years	10 [2; 14]	6 [2; 19]	9 [5; 17]	*p* > 0.05
Age at onset, years	50 [45; 63]	57 [45; 67]	57 [45; 74]	*p* > 0.05
**Baseline (at the beginning of treatment course)**				
**Scores by:**				
PANSS-Pos	27 [24; 33]	24 [17; 26]	26 [21; 29]	*p* > 0.05
Hallucinations	**4 [2; 5]**	**2 [1; 4]**	**5 [1; 6]**	***p*** ^**1–2**^ **= 0.046**
				***p*** ^**2–3**^ **= 0.007**
Grandiosity	**3 [2; 4]**	**1 [1; 3]**	**1 [1; 1]**	***p*** ^**1–2**^ **= 0.042**
				***p*** ^**1–3**^ **= 0.001**
Suspiciousness	**5 [4; 7]**	**4 [3; 5]**	**5 [3; 5]**	***p*** ^**1–2**^ **= 0.039**
PANSS-Neg	23 [18; 27]	17 [14; 25]	21 [17; 24]	*p* > 0.05
Blunted affect	**4 [3; 5]**	**3 [1; 4]**	**3 [2; 4]**	***p*** ^**1–2**^ **= 0.042**
Poor rapport	**3 [3; 4]**	**1 [1; 4]**	**3 [2; 3]**	***p*** ^**1–2**^ **= 0.024**
PANSS-Psy	49 [45; 59]	50 [44; 55]	50 [45; 56]	*p* > 0.05
PANSS total	99 [87; 120]	93 [85; 101]	95 [86; 110]	*p* > 0.05
CGI-S	5 [4; 6]	5 [4; 5]	5 [4; 6]	*p* > 0.05
HAMD-17 total	19 [15; 24]	19 [14; 26]	22 [19; 25]	*p* > 0.05
MMSE	27 [24; 28]	25 [23; 28]	24 [22; 27]	*p* > 0.05
CDSS total	8 [5; 11]	9 [4; 12]	8 [6; 11]	*p* > 0.05
Number (portion, %) of patients with depression	12 (75%)	14 (70%)	18 (78%)	*p* > 0.05
**At 28-th day of the treatment course**				
**Scores by:**				
PANSS-Pos	17 [12; 20]	13 [10; 15]	15 [14; 18]	*p* > 0.05
Hallucinations	**2 [1; 3]**	**1 [1; 2]**	**3 [1; 3]**	***p*** ^**1–2**^ **= 0.040**
Grandiosity	**2 [2; 3]**	**1 [1; 2]**	**1 [1; 1]**	***p*** ^**1–3**^ **= 0.006**
Suspiciousness	3 [2; 3]	3 [1; 3]	3 [2; 3]	*p* > 0.05
PANSS-Neg	18 [13; 22]	13 [11; 18]	17 [14; 20]	*p* > 0.05
Blunted affect	3 [2; 4]	2 [1; 3]	3 [1; 3]	*p* > 0.05
Poor rapport	**3 [2; 3]**	**1 [1; 2]**	**2 [1; 3]**	***p*** ^**1–2**^ **= 0.040**
PANSS-Psy	36 [34; 43]	32 [28; 37]	34 [30; 41]	*p* > 0.05
PANSS total	72 [56; 81]	62 [52; 68]	67 [59; 78]	*p* > 0.05
CGI-S	3 [2; 4]	2 [1; 3]	3 [2; 3]	*p* > 0.05
HAMD-17 total	9 [3; 11]	6 [3; 10]	8 [5; 11]	*p* > 0.05
MMSE	29 [28; 29]	28 [25; 30]	26 [24; 28]	*p* > 0.05

Medians and [25, 75%-Quartiles] are given in the table. Significant differences are given in bold.

We have introduced a binary parameter (equal to “yes” or “no”) in the database reflecting the side effect, registered before the beginning of treatment, namely extrapyramidal side-effects, such as hypokinesia, expressionless face, lack of associated movements when walking, in line with rigidity, coarse tremor; anticholinergic side-effects, such as dry mouth, reduced sweating, constipation, blurred vision; antiadrenergic side-effect, such as postural hypotension and metabolic syndrome.

The control group (*n* = 38), all volunteers without psychiatric or neurological diagnosis (they did not have symptoms or psychometric assessments), was age and gender matched with the total patients’ group, and consisted of 34 females and 4 males (51–84 years old; median age 61 [53; 68]).

### 2.2 Measurement of blood biochemical parameters

Blood samples were collected and treated as described previously (32). Isolation of platelets and erythrocytes, preparation of protein extracts from these cells, and determination of enzymatic activity of glutamate-, energy-, and glutathione antioxidant metabolism enzymes, namely, glutamate dehydrogenase (GDH), cytochrome c-oxidase (COX), glutathione reductase (GR), glutathione-S-transferase (GST) in the prepared extracts were carried out as described previously (32). Enzymatic activities of GDH and COX were measured in extracts from platelets only; activities of GR and GST were measured in extracts from platelets (GRpl, GSTpl), and in extracts from erythrocytes (GRer, GSTer) using spectrophotometric methods as described previously (32–34). Protein concentration was determined by the Lowry method using a Bio-Rad DC Protein Assay (United States) and bovine serum albumin (Sigma-Aldrich, United States) as a protein standard. After determining the protein concentration, the specific activity of each enzyme (U/mg) was calculated. The enzymatic activities were assessed once in the control group and twice in patients, as mentioned above.

### 2.3 Statistical analysis

For statistical analysis data of patient psychometric examination and measurements of enzymatic activities, the “nonparametric analysis” module of the Statistica 8.0 software (StatSoft) was used. Mann–Whitney *U*-test, Wilcoxon pairwise comparison method, Spearman’s rank correlation coefficients, Kruskal-Wallis test, and χ^2^-test (Хi-square test) were used for assessing the significance of differences, changes in parameters, and correlations between them. Differences and correlations were considered significant at *p* < 0.05.

*Post hoc* comparative analysis of blood biochemistry was done in patients with LOP treated with neuroleptics plus antidepressants and adjunctive medicines, as well as in patients treated with neuroleptics plus antidepressants medicines without adjunctive drugs. For the post-hoc analysis of biochemical data for patients with LOP we have chosen activities of blood enzymes involved in glutathione antioxidant and glutamate metabolism, based on our previous successful use of these parameters in stratification of patients in old age psychiatry (31).

## 3 Results

### 3.1 Patients’ subgroups

Based on prescribed adjunctive therapy, patients were divided into three subgroups: the first subgroup received only antipsychotics and antidepressants without adjunctive therapy (Gr1, *n* = 16), whereas two other subgroups were prescribed adjunctive medicines, such as either EMHS (Gr2, *n* = 20) or other drugs including citicoline, cerebrolysin, cortexin, actovegin, gliatilin (Gr3, *n* = 23).

Although there were no significant differences in baseline PANSS total scores between Gr1, Gr2, and Gr3 groups (Table 1), the scores by PANSS-Pos subscale items for hallucinations, grandiosity, and suspiciousness and PANSS-Neg scores for blunted affect and poor rapport were significantly lower in Gr2 than in Gr1 (Table 1). As for Gr3, there was a significant difference with Gr1 in scores by PANSS-Pos items for grandiosity, suspiciousness, and PANSS-Neg scores for blunted affect (all lower in Gr3). Patients in Gr2 group, compared with Gr3 group, had lower PANSS-Pos scores in the subscale items for hallucinations. The scores for this symptom were the lowest in Gr2 among all groups of patients.

Patients assigned to Gr2 had some additional clinical symptoms. Thus, they displayed hypochondria, preoccupation with their physical condition, and somatic concern. Besides, cerebellar atrophy seen on computer tomography (CT) of the brain (CT evidence of cerebellar atrophy was obtained only once, before the prescription) and manifested as ataxia, uncertain gait and falls served as a basis for assignment of a patient to Gr2 (31).

Although before the start of the study all the patients (being outpatients) might receive various psychotropic therapies, after their hospitalization and inclusion in the study all the patients received such antipsychotic therapy, that Gr1, Gr2 and Gr3 patients did not significantly differ (Chi-square test, *p* > 0.05) in the most commonly prescribed antipsychotics, such as olanzapine and haloperidol (Table 2). Also, no significant between-group differences were found for antidepressant therapy with fluvoxamine, the most commonly prescribed antidepressant, *p* > 0.05 by Chi-square test (Table 2).

**Table 2:** Numbers and percentages of patients, to whom the psychotropic medicines were prescribed, and ranges of their dosages (in mg per day).

Medicines	Groups, patient numbers, (%)		
	Gr1 (*n* = 16)	Gr2 (*n* = 20)	Gr3 (*n* = 23)
Olanzapine dose, mg	8 (50%)	10 (50%)	12 (52%)
	5–15	5–12.5	5–15
Haloperidol dose, mg	4 (25%)	8 (40%)	6 (26%)
	5–15	5–12.5	5–15
Quetiapine dose, mg	3 (19%)	4 (20%)	6 (26%)
	50–200	50–200	50–200
Risperidone dose, mg	3 (19%)	4 (20%)	6 (26%)
	1–6	1–6	1–6
Fluvoxamine dose, mg	7 (44%)	9 (45%)	9 (39%)
	50–150	25–125	25–150

The sum of patients’ numbers, to whom each the medicine was prescribed, exceeds the total number of patients in group because some patients were switched from one antipsychotic medicine to another during the treatment course or they received two antipsychotics (for instance, olanzapine and haloperidol were prescribed to two patients in Gr1, and three patients in each group Gr2 and Gr3).

A significant between-group differences were found for the frequency of reported side effects assessed at the beginning of treatment. When the groups were pairwise compared, significant differences were found between Gr1/Gr3, and Gr1/Gr2 (Table 3). Thus, the Gr1 was characterized by prominent severity of productive symptoms, which was a contraindication for adjuvant treatment. The anticholinergic side effects prevailed in Gr1. The Gr2 was characterized by prevalence of antidopaminergic effects and the Gr3—antiadrenergic effects and risk of metabolic syndrome.

**Table 3:** Frequency of side effect appearance during psychotropic treatment.

Presence of side effects	Groups, patient numbers, %			Yates’s corrected Хi-square, *p-*level
	Gr1 (*n* = 16)	Gr2 (*n* = 20)	Gr3 (*n* = 23)	
No	4 (25%)	8 (40%)	12 (52%)	Gr1/Gr3
				14.28, 0.0002
Yes	12 (75%)	12 (60%)	11 (48%)	G1/Gr2
				4.47, 0.03

### 3.2 Analysis of blood biochemistry in Gr1, Gr2, and Gr3

**Figure 1:**
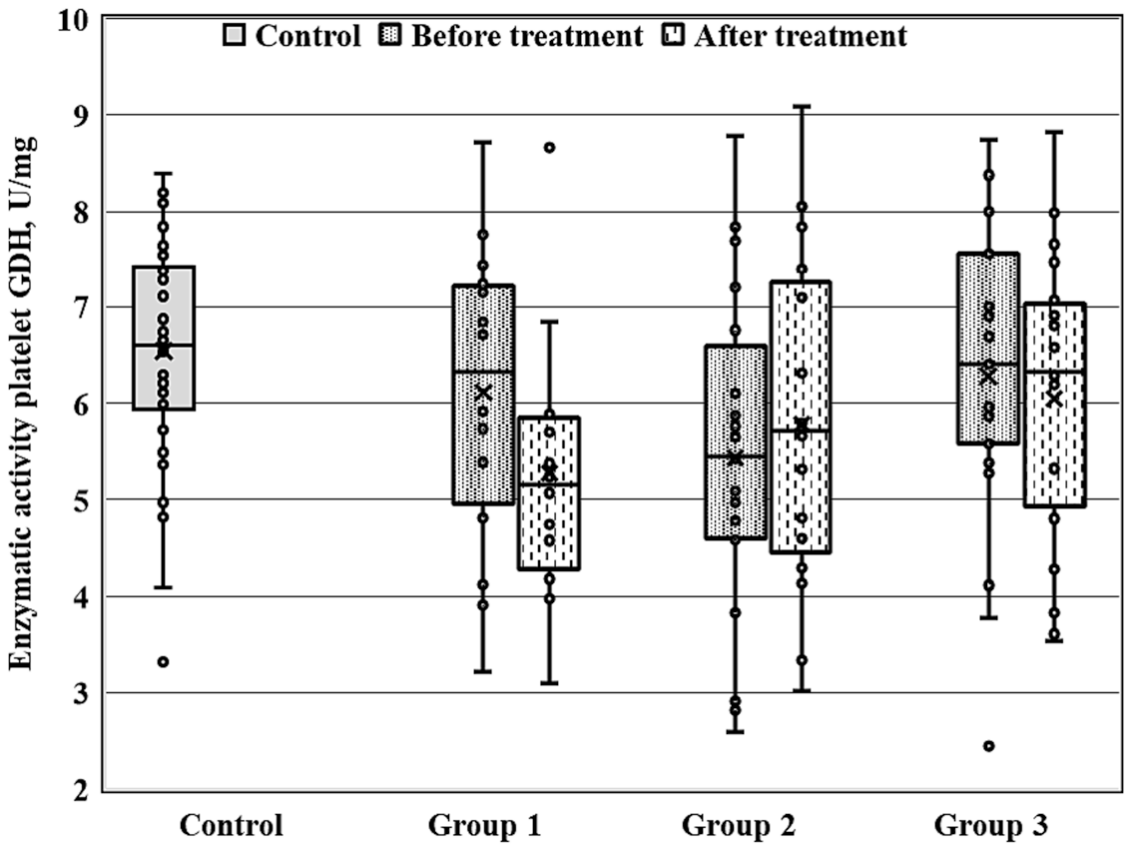
Enzymatic activity of GDH measured before and after the treatment course in Gr1 (Group 1), Gr2 (Group 2), and Gr3 (Group 3) patients, as well as in the matched control group. Baseline GDH activity was significantly decreased in Gr2 (*p* < 0.005 by Mann–Whitney *U*-test) in comparison to the control group.

**Figure 2:**
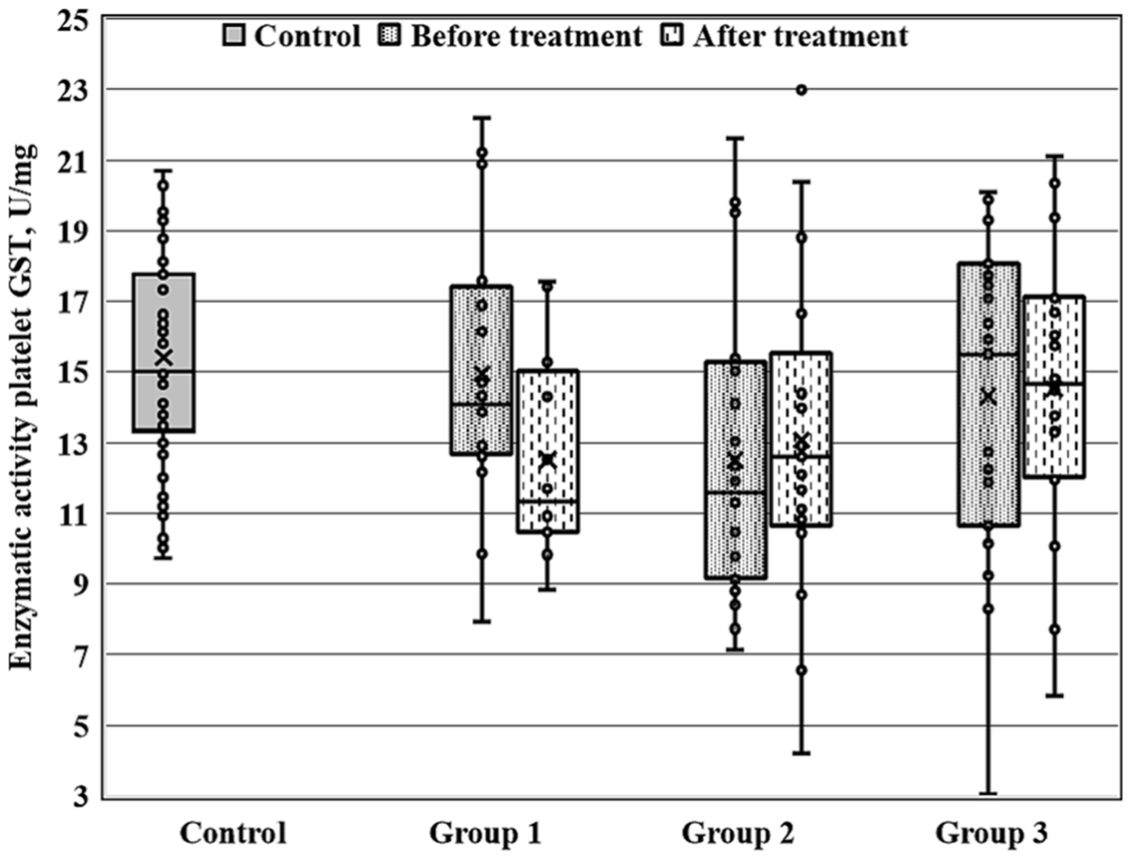
Enzymatic activity of platelet GST (GSTpl) measured before and after the treatment course in Gr1 (Group 1), Gr2 (Group 2), and Gr3 (Group 3) patients, as well as in the matched control group. Baseline GSTpl activity was significantly decreased in Gr2 (*p* < 0.005 by Mann–Whitney *U*-test) in comparison to the control group.

**Figure 3:**
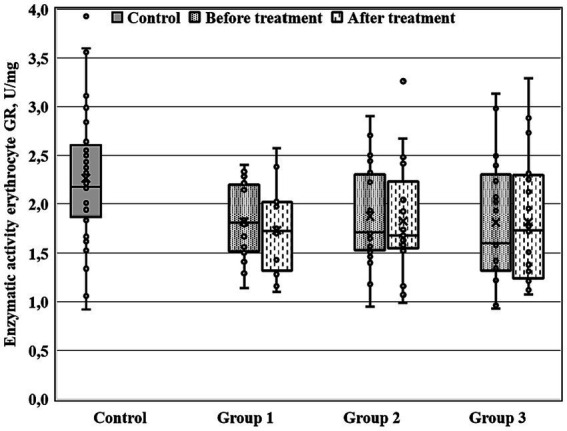
Enzymatic activity of erythrocyte GR (GRer) measured before and after the treatment course in Gr1 (Group 1), Gr2 (Group 2), and Gr3 (Group 3) patients, as well as in the matched control group. GRer was uniformly decreased as at baseline, as after the treatment course in all three patient groups in comparison with the control group (*p* < 0.01 by Mann–Whitney *U*-test for every group).

There were significant between-group differences at 28-th day of the treatment course for three enzymatic activities (GDH, GSTpl, and GRer), whereas only GRer differed between the regarded groups at baseline (Table 4; Figures 1–3). This data suggested further analysis of the biochemical parameters in patients and controls.

**Table 4:** Enzymatic activities for glutamate dehydrogenase (GDH), glutathione reductase (GR), and glutathione-S-transferase (GST) in platelets and erythrocytes compared by Kruskal-Wallis test in control group and three patients’ groups (Gr1, Gr2, Gr3).

Enzymatic activity	Baseline		At 28-th day of the treatment course	
	Н	*p*-level	Н	*p*-level
COX	5.18	0.159	5.27	0.153
GDH	7.62	0.054	**9.61**	**0.022**
GRpl	2.80	0.423	4.68	0.197
GSTpl	7.61	0.055	**8.77**	**0.033**
GRer	**10.27**	**0.016**	**10.78**	**0.013**
GSTer	0.81	0.847	0.91	0.823

Significant differences are given in bold.

Figures 1–3 show the enzymatic activities of GDH, GSTpl, and GRer measured at baseline and at 28-th day of the treatment course in Gr1 (without adjunctive therapy), Gr2 (with adjunctive EMHS), and Gr3 patients (with adjunctive citicoline, cerebrolysin, cortexin, actovegin, or gliatilin), as well as in the matched control group.

As seen in Table 4 and Figure 3, in comparison with the control group, GRer was uniformly decreased at baseline, as after the treatment course in all three patient groups (*p* < 0.01 by Mann–Whitney U-test for every group). However, several between-group differences were found for other biochemical parameters when groups of patients were compared pairwise. So, *baseline* GDH and GSTpl activities were significantly decreased in Gr2 (but not in Gr1 or Gr3) compared to control subjects (*p* < 0.005 for each pairwise comparison of the groups with the control group; Figures 1, 2). After the treatment course GDH activity in Gr2 returned to the control value, and it was undistinguishable from the control one (*p* > 0.05), unlike Gr1, whereas GDH declined after antipsychotic treatment course without adjunctive medicine to the level significantly lower than that of the control group (*p* < 0.002; Figure 1).

When biochemical parameters were compared at baseline and after the treatment by Wilcoxon matched pairs test, only Gr1 (treated without adjunctive medicines) demonstrated a significant decrease in platelet GST after the treatment course (*p* < 0.006), and, as a result, the GST activity after the treatment course became significantly decreased in Gr1 in comparison with control group (*p* < 0.02, by Mann–Whitney *U*-test).

### 3.3 Correlations between blood biochemical parameters and clinical assessment scores

Different significant links between biochemical parameters (measured at *baseline*) and scores by clinical scales PANSS, HAMD-17, CDSS and CGI-S were found in Gr1 (treated with psychotropic medicines without adjuncts), Gr2 (with adjunctive EMHS), and Gr3 (with adjunctive citicoline, cerebrolysin, cortexin, actovegin, or gliatilin; Table 5).

**Table 5:** Correlations between baseline (measured before the treatment) biochemical parameters (activity of GSRer, GDH, GRpl) and scores by clinical scales (PANSS and its subscales, CGI-S, HAMD-17), measured before and after the treatment course.

Measurement of scores by scales before or after treatment course	Gr1			Gr2			Gr3		
	Correlating parameters	R	*p*	Correlating parameters	R	*p*	Correlating parameters	R	*p*
Before	GSTer and PANSS-tot	0.62	0.010	GSTer and PANSS-tot	0.60	0.005			
	GSTer and PANSS-Pos	0.46	0.075	GSTer and PANSS -Pos	0.53	0.016			
	GSTer and PANSS-neg	0.61	0.012						
	GSTer and PANSS-psy	0.54	0.032	GSTer and PANSS-psy	0.52	0.017			
	GSTer and CGI-S	0.51	0.029	GSTer and CGI-S	0.59	0.008			
							GRpl and PANSS-pos	0.50	0.014
							GRpl and CDSS	−0.43	0.039
After				GSTer and PANSS-psy	0.45	0.049			
				GSTer and CGI-S	0.47	0.038			
	GDH and PANSS-tot	0.63	0.028	GDH and PANSS-tot	−0.44	0.042			
	GDH and PANSS-pos	0.84	0.001						
	GDH and HAMD-17	0.66	0.021						
	GDH and CGI-S	0.66	0.021	GRpl and CGI-S	−0.63	0.004			
							GRpl and HAMD-17	−0.54	0.009

Gr1 particularly demonstrated several unique associations between biochemical parameters and clinical assessments. For instance, GSTer activity was significantly linked with *baseline* PANSS total scores, PANSS-neg and PANSS-psy scores, and CGI-S scores (Table 5). Besides, significant associations were found between GDH activity and clinical assessments *after the treatment* course, such as PANSS total scores and PANSS-pos scores (Table 5). Unique links for Gr1 were found for GDH activity with HAMD-17 and CGI-S scores evaluated *after the treatment* course (Table 5).

In Gr2 group, like in Gr1, there were significant correlations of GSTer with *baseline* PANSS total and PANSS-psy scores (Table 5). A significant link between GSTer and *baseline* CGI-S scores (R = 0.59, *p* = 0.008) was found in Gr2, similarly to those found in Gr1. For Gr2, there was a significant negative association between GDH and PANSS total scores *after the treatment* (Table 5). Interestingly, this was a negative correlation, in contrast to the positive relationship between the same parameters found in Gr1 group. Unlike two other groups of patients, Gr2 demonstrated unique significant links of GSTer with PANSS-psy scores and CGI-S scores (Table 5), both clinical parameters evaluated *after the treatment* course. Also, significant link of GRpl with CGI-S scores, evaluated *after the treatment* course was found in Gr2 (Table 5).

Gr3 group demonstrated also unique links: GRpl was correlated with *baseline* PANSS-pos scores, CDSS scores, as well as HAMD-17 scores evaluated *after the treatment course* (Table 5).

## 4 Discussion and conclusion

The results of our study demonstrate the validity of adjunctive neuroprotective medicines use in addition to antipsychotic plus antidepressant therapy in distinct subgroups of patients suffering with LOP, and especially those who have prominent side effects, namely antidopaminergic movement side effects, antiadrenergic effects, and metabolic syndrome, accompanying psychotropic treatment. In present work we have succeeded in achieving the target of our study. Namely, we have found distinct clinical and biochemical parameters which enable discriminating and separating subgroups of patients to whom the prescribing of the additional pharmacotherapy with EMHS, citicoline, cerebrolysin, cortexin, actovegin, gliatilin (choline alfoscerate) is beneficial and safe, in agreement with previous findings (6, 31). Particularly, we have reported that baseline clinical parameters such as scores by PANSS items (hallucinations, grandiosity, suspiciousness, blunted affect, poor rapport) are lower in patients with LOP to whom the adjunctive therapy, especially with EMHS, is to be prescribed. As for biochemical parameters, only GRer differed between the analyzed groups at baseline, although significant between-group differences were observed for three enzymatic activities (GDH, GSTpl, and GRer) after the treatment course. This suggests that there are additional influences on the activity of blood enzymes in patients with LOP. Furthermore, we have confirmed the earlier pilot findings (6) on significantly lower frequency of the side effects’ arising in subgroups of patients with LOP to whom the adjunctive pharmacotherapy is prescribed in addition to antipsychotics and antidepressants.

We have also found several biochemical parameters, such as GDH, GSTer, and GRpl, which being measured at baseline could have a predicting value, different in various groups of patients. Measurement of these baseline biochemical parameters (linked with scores by clinical scales after the treatment course), could facilitate predicting the efficacy of the antipsychotic pharmacotherapy in selected groups of patients.

Our study compares favorably with those available in the literature by careful selection of patients and the use of several psychometric scales to assess the condition before and after the course of therapy. We used a standardized rating scales for assessing the condition of patients with LOP, while patients with organic brain pathology and dementia syndrome were excluded based on their clinical history and assessment, including brain MRI scan. The use of the MMSE was of an auxiliary nature, enabling to assess the dynamics of cognitive functions during treatment, although low scores at baseline were not the main criterion for assessing the degree of cognitive decline due to the severity of psychotic disorders makes it difficult to perform cognitive tests like MMSE. This was an explanation why one of the exclusion criteria from our study was moderate to severe memory impairment (overall score on the MMSE ≤ 25) *at the end of the treatment course against the background of a decrease in the severity of psychotic symptoms and improvement in their mental condition*. At the same time, various cognitive impairments in some patients were of a diffuse nature at the level of age-associated decline and were not regarded as a criterion for exclusion from the study.

It was important for us to identify and treat depression in patients with LOP (schizophrenia). Depressive symptoms are recognized as a part of the symptom pattern in patients with schizophrenia (their frequency achieves 7% to 80%) (35, 36). Such a scatter of data for the depression symptoms’ frequency, to some extent, may be due to the patients being assessed at different stages of the disease, diagnostic uncertainty, the lack of a unified methodological approach and algorithm for using certain scales to identify depressive symptoms in schizophrenia (37). Nevertheless, depression can develop in every form and at every stage of the schizophrenia course, that is not surprising, given the pathophysiology (biological, neurochemical background) of the main symptoms of affective and psychotic disorders (38, 39). For the purposes of clinical studies of schizophrenia, the Calgary Depression Scale for Schizophrenia (CDSS) was developed and is still used (including our study), allowing in some cases to differentiate affective and negative symptoms (40). In our study, at the baseline, depression by CDSS was detected in ~77% of patients. Depressive symptoms were often associated with a response to psychosis, immediately arising in the structure of the psychotic episode, and they usually reduced along with psychotic symptoms [19, 26]. In addition to the CDSS scale, we used the Hamilton scale (HDRS-17), the standard for determining the therapy efficacy in the treatment of depressive disorders, for quantification the severity of depressive symptoms, and the scores obtained using these scales correlated with biochemical parameters measured in our study (41).

Last but not least, our present study was devoted to adjunctive therapy in POL. There are very few studies on the pharmacotherapy of LOP (late-onset schizophrenia), and most of them have not been included in the Cochrane review due to their poor design (42). The use of antipsychotics, and to a more restricted degree antidepressants, is associated with an increased risk for several somatic diseases, including metabolic syndrome, musculoskeletal and renal diseases, as well as movement and seizure disorders, and obesity (43). Not surprisingly, the side effects are associated with poor patients’ compliance, especially evident in the aging population (44). Since the clinical utility of approved treatments for tardive dyskinesia is unclear (45), new approaches to overcome the problem would be beneficial for patients suffering from side effects of antipsychotics.

In our study we have clearly observed between-group difference with lesser degree of side effects accompanying psychotropic treatment in the group treated with EMHS and in the group treated with citicoline, cerebrolysin, cortexin, actovegin, gliatilin (choline alfoscerate) as adjunctive medicines. Since the patient groups did not differ in the psychotropic therapy, we can assume that the decrease in the severity of side effects of the therapy at the end of the study in two groups, receiving the additional medicines, may be due precisely to their action. Previous studies have demonstrated general validity of EMHS for patients with schizophrenia (11, 12), whereas the present study represents the first attempt of stratified approach to patients assigned to subgroups with different adjunctive therapy in addition to standard antipsychotic and antidepressant therapy prescribed in LOP. We have succeeded not only in finding clinical features for selecting patients to whom the treatment with EMHS (Gr2) as an adjunctive medicine is favorable, but also in revealing their blood biochemical features discriminating them from other patients with LOP. EMHS, the antioxidant and anti-hypoxia medicine, is available not only in the form of intravenous injections, but also as an oral preparation, thus widening the EMHS for outpatients as well. The easy and relatively not expensive measured biochemical parameters used in the present study enabled to stratify patients and select, a subgroup with the most favorable effect from adjunctive therapy with EMHS. These parameters may help, in addition to clinical features, not only to select patients for adjunctive medicine prescribing, but may also have a predictive value. Since baseline GDH significantly correlated with PANSS scores measured after the treatment course (positively correlated in Gr1, but negatively in Gr2), the baseline GDH level proved to have a predictive meaning in Gr1, as well as in Gr2. Also, baseline GRpl and GSTer activities may have predictive value in Gr2, because they were linked with PANSS or CGI-S scores measured after the treatment course.

For patients in the group, treated with various adjunctive medicines other than EMHS, there was also a unique negative correlation between *baseline* GRpl and HAMD-17 scores post *treatment*, meaning that the higher the GRpl initial activity was, the lesser depression scores were after the treatment course. In this group, various adjunctive medicines were applied, possibly exhibiting different neuroprotective effects, and their efficacy has been already compared in animal model (16). The comparative investigation of citicoline, cerebrolysin, cortexin, actovegin, and gliatilin in clinical practice would represent interest in future.

One of the limitations of our study is the low number of patients treated with individual adjunctive medicines. We have, therefore, to combine them into one group [Gr3, treated with citicoline, cerebrolysin, cortexin, actovegin, gliatilin (choline alfoscerate)]. Hence, taking into account our present encouraging results, we plan to enlarge the groups in future. When conducting a further study on a larger sample with an appropriate design, the results would be more conclusive and might have scientific and practical interest for assessing the possibility of using antioxidant and “metabolic” medicines as additional therapy for late-onset schizophrenia.

We report here not only selective clinical and biochemical criteria for prescribing adjunctive medicine in LOP, but we also describe some predictive biomarkers for the selected groups. The results obtained in the present study may serve as a background for trials of other antioxidants and neuroprotective substances, either synthetic or of natural origin as adjunctive medicines in psychiatry to overcome problem of side effects associated with psychotropic medicines.

## Data availability statement

The raw data supporting the conclusions of this article will be made available by the authors, without undue reservation.

## Ethics statement

The studies involving humans were approved by Oleichik, Igor V., Department of Endogeneous Mental Disorders, Mental Health Research Centre. The studies were conducted in accordance with the local legislation and institutional requirements. The participants provided their written informed consent to participate in this study.

## Author contributions

IB: Conceptualization, Methodology, Writing – review & editing. OS: Data curation, Formal analysis, Software, Validation, Writing – original draft. VS: Data curation, Investigation, Methodology, Project administration, Writing – original draft. ET: Data curation, Investigation, Visualization, Writing – original draft. TP: Data curation, Visualization, Writing – original draft. VP: Investigation, Writing – original draft. GB: Supervision, Writing – original draft.
